# miR-30 Family Controls Proliferation and Differentiation of Intestinal Epithelial Cell Models by Directing a Broad Gene Expression Program That Includes SOX9 and the Ubiquitin Ligase Pathway*[Fn FN2]

**DOI:** 10.1074/jbc.M116.733733

**Published:** 2016-06-03

**Authors:** Bailey C. E. Peck, John Sincavage, Sydney Feinstein, Amanda T. Mah, James G. Simmons, P. Kay Lund, Praveen Sethupathy

**Affiliations:** From the ‡ Curriculum in Genetics & Molecular Biology,; the Departments of § Genetics,; ¶ Nutrition, and; ‖ Cell Biology and Physiology, and; the ** Lineberger Comprehensive Cancer Center, University of North Carolina, Chapel Hill, North Carolina 27599

**Keywords:** differentiation, gene regulation, intestinal epithelium, microRNA (miRNA), proliferation

## Abstract

Proliferation and differentiation of intestinal epithelial cells (IECs) occur in part through precise regulation of key transcription factors, such as SOX9. MicroRNAs (miRNAs) have emerged as prominent fine-tuners of transcription factor expression and activity. We hypothesized that miRNAs, in part through the regulation of SOX9, may mediate IEC homeostasis. Bioinformatic analyses of the *SOX9* 3′-UTR revealed highly conserved target sites for nine different miRNAs. Of these, only the miR-30 family members were both robustly and variably expressed across functionally distinct cell types of the murine jejunal epithelium. Inhibition of miR-30 using complementary locked nucleic acids (LNA30bcd) in both human IECs and human colorectal adenocarcinoma-derived Caco-2 cells resulted in significant up-regulation of *SOX9* mRNA but, interestingly, significant down-regulation of SOX9 protein. To gain mechanistic insight into this non-intuitive finding, we performed RNA sequencing on LNA30bcd-treated human IECs and found 2440 significantly increased genes and 2651 significantly decreased genes across three time points. The up-regulated genes are highly enriched for both predicted miR-30 targets, as well as genes in the ubiquitin-proteasome pathway. Chemical suppression of the proteasome rescued the effect of LNA30bcd on SOX9 protein levels, indicating that the regulation of SOX9 protein by miR-30 is largely indirect through the proteasome pathway. Inhibition of the miR-30 family led to significantly reduced IEC proliferation and a dramatic increase in markers of enterocyte differentiation. This in-depth analysis of a complex miRNA regulatory program in intestinal epithelial cell models provides novel evidence that the miR-30 family likely plays an important role in IEC homeostasis.

## Introduction

The intestinal epithelium is vital for a wide range of physiological functions, including pathogen defense, nutrient absorption, and metabolic homeostasis. It is also the most rapidly renewing tissue in the body, with cellular turnover occurring every 3–5 days in humans. This rate is dependent on the stability of intestinal epithelial stem cells, which give rise to transit amplifying progenitor cells that go on to differentiate into various types of mature IECs, such as enterocytes, enteroendocrine cells, Paneth cells, and goblet cells. Precise regulation of gene expression in these cell types is vital for the proper balance between proliferation and differentiation in the intestinal epithelium.

miRNAs^2^ are prominent members of gene regulatory networks and are known for their roles in buffering and fine-tuning target gene expression (1–3). However, their functions in the intestinal epithelium are understudied, particularly in relation to other metabolic tissues such as liver and muscle. The very few published studies suggest that miRNAs are likely important in shaping intestinal epithelial architecture, barrier function, and proliferation (4–6). More recently, it has been proposed that miRNAs likely produced from IECs may regulate resident microbial communities (7). Because miRNAs are attractive therapeutic targets in an increasing array of disorders (8), identifying specific miRNAs and their regulatory pathways that govern key physiological processes in the intestine is an important step toward the development of novel, effective therapeutic targets for gastrointestinal diseases associated with altered intestinal epithelial proliferation and differentiation.

To identify miRNAs potentially involved in intestinal epithelial homeostasis, we started by *in silico* prediction of miRNAs with putative target sites in *SOX9*, which encodes a transcription factor that is well known for its regulatory role in gastrointestinal biology. Like other members of the SRY box family of transcription factors, SOX9 is tightly regulated by a complex network of transcriptional, post-transcriptional (9), and post-translational (10) modifiers. It is known to regulate proliferation and differentiation of diverse stem and progenitor cells, including but not limited to gonad (11), chondrocyte (12), neural crest (13), lung (14), pancreas (15), and intestinal epithelium (14–17). The functional effect of Sox family members in general is frequently described as dosage-dependent, with relative expression levels driving either cellular renewal or differentiation (18, 19). Notably, varying levels of *Sox9* have been shown to mark functionally distinct cell types of the mouse intestinal epithelium. Accordingly, a transgenic reporter mouse (*Sox9-EGFP*) has been developed to identify and isolate both differentiated cell types and actively cycling intestinal epithelial stem cells and progenitors based on the levels of cellular EGFP expression driven by the *Sox9* promoter (20–24).

SOX9 is not uniquely expressed in IECs, and a few studies to date have assessed miRNA targeting of *SOX9* in other tissues. For example, miR-145 has been shown to target *SOX9* in various cancer subtypes (25, 26) and chondrocytes (27). Both miR-145 and miR-495 target *SOX9* in mesenchymal stem cells (9, 28), and miR-101 targets *SOX9* in hepatocellular carcinoma (29). Because both miRNA expression and mRNA 3′-UTR usage can vary across cell types and conditions, these findings are not necessarily generalizable to the intestinal epithelium. To date no study has investigated miRNA-mediated regulation of *SOX9* in the context of IECs. More importantly, roles of specific miRNAs in the control of intestinal epithelial proliferation and differentiation are poorly characterized. In this study, we work toward bridging this knowledge gap using *in silico*, *in vitro*, and *in vivo* analyses.

## Results

### 

#### 

##### miR-30 Is Predicted to Target SOX9 and Is Robustly Expressed in the Intestinal Epithelium

**FIGURE 1.**
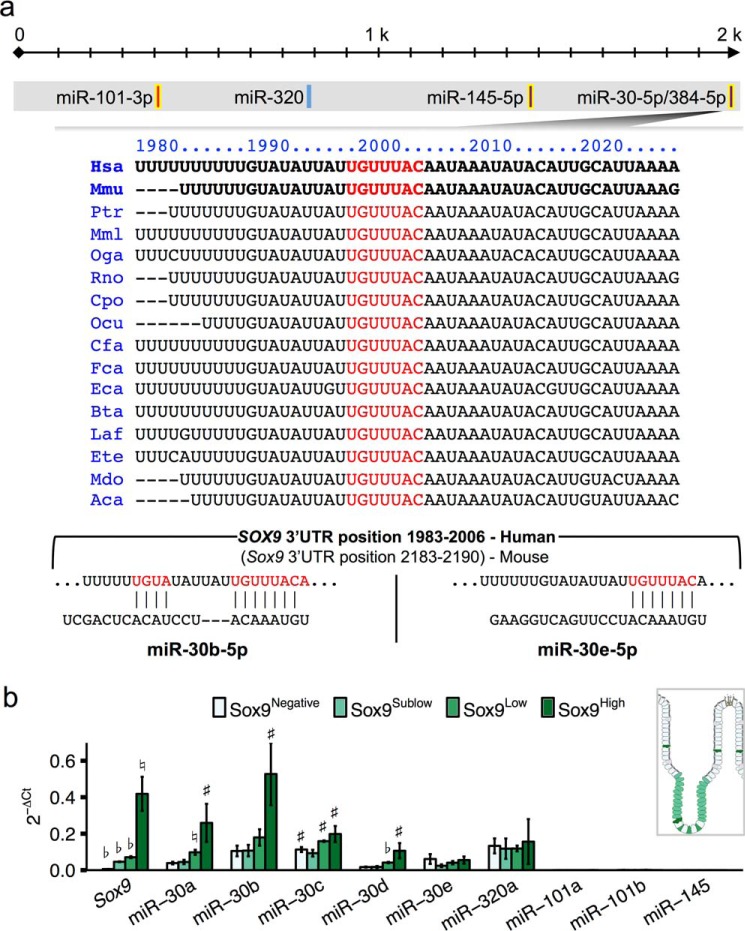
**miR-30 is predicted to target the 3′-UTR of *SOX9* and is differentially expressed across functionally distinct cell types of the intestinal epithelium.** *a*, cartoon depicting the *SOX9* 3′-UTR. miRNAs with predicted target sites conserved between mouse and human are labeled. Below, we show the conservation of the predicted miR-30 target site (*red text*) across various species (TargetScan6.2). miR-30b and miR-30e targeting are shown in detail with predicted base paring colored in *red. b*, RT-PCR of *Sox9* and miRNAs with predicted target sites in the *Sox9* 3′-UTR across functionally distinct mouse jejunal intestinal epithelial cells (*n* = 2–4). Significance was determined by Student's two-tailed paired *t* test relative to non-sorted intestinal epithelial cells. ♭, *p* < 0.001; ♮, *p* < 0.01; #, *p* < 0.05.

We carried out a bioinformatic strategy using TargetScan6.2 (30–33) to predict miRNA target sites in the *SOX9* 3′-UTR that are conserved between mouse and human. We identified putative target sites for nine miRNA families. To narrow this list of possible miRNA regulators of *SOX9* in the intestinal epithelium, we analyzed the only data set of publically available small RNA sequencing data from mouse intestinal mucosa (4). Only four miRNA families were expressed at a minimum of 10 reads/million mapped: miR-145, miR-101, miR-320, and miR-30 (Fig. 1*a*). Of these, miR-30 has the strongest predicted base pairing with *SOX9*, consisting of an 8-mer seed as well as supplementary 3′-end pairing for two of the family members. Moreover, the miR-30 target site and flanking ∼15 bases are highly conserved among most mammals including human, rodent, dog, opossum, and horse, as well as distant vertebrates such as lizard.

Because the intestinal mucosa includes diverse cell types not limited to epithelia, we next sought to evaluate the expression of the members of these four miRNA families across four main epithelial cell types. Specifically, we sorted functionally distinct IECs by FACS from the jejunum of female conventionally raised *Sox9-EGFP* mice. This model allows for the isolation of four populations based on cellular EGFP, including enteroendocrine cells (Sox9^High^), intestinal epithelial stem cells (Sox9^Low^), transit amplifying cells (Sox9^Sublow^), and differentiated enterocytes and Paneth and goblet cells (Sox9^Negative^). We then performed RT-PCR for each of the four miRNA families across each IEC population. miR-101 and miR-145 were very lowly expressed, indeed barely detected, in any cell type of the intestinal epithelium (Fig. 1*b*). It is worth noting that although miR-145 was reported to have robust expression in the study by McKenna *et al.* (4) of the entire intestinal mucosa, it was recently demonstrated that miR-145 is specific to mesenchymal cells in the intestine (34). By using FACS, we obtain a highly pure epithelial population, whereas the earlier data from McKenna *et al.* (4) were generated using an intestinal scraping method, which could lead to some mesenchymal, lymphatic, and/or vascular contamination. Based on these differences, we conclude that it is likely that both miR-145 and miR-101 are robustly expressed in a non-epithelial mucosal tissue, but not in IECs. In contrast, members of the miR-30 family and miR-320a showed robust expression in IECs (Fig. 1*b*). Moreover, only miR-30 family members exhibited differential expression across functionally distinct IECs, leading us to select this miRNA family for follow-up analyses.

##### Knockdown of miR-30 in Vitro Results in Increased SOX9 mRNA Expression, but Decreased Levels of SOX9 Protein

**FIGURE 2.**
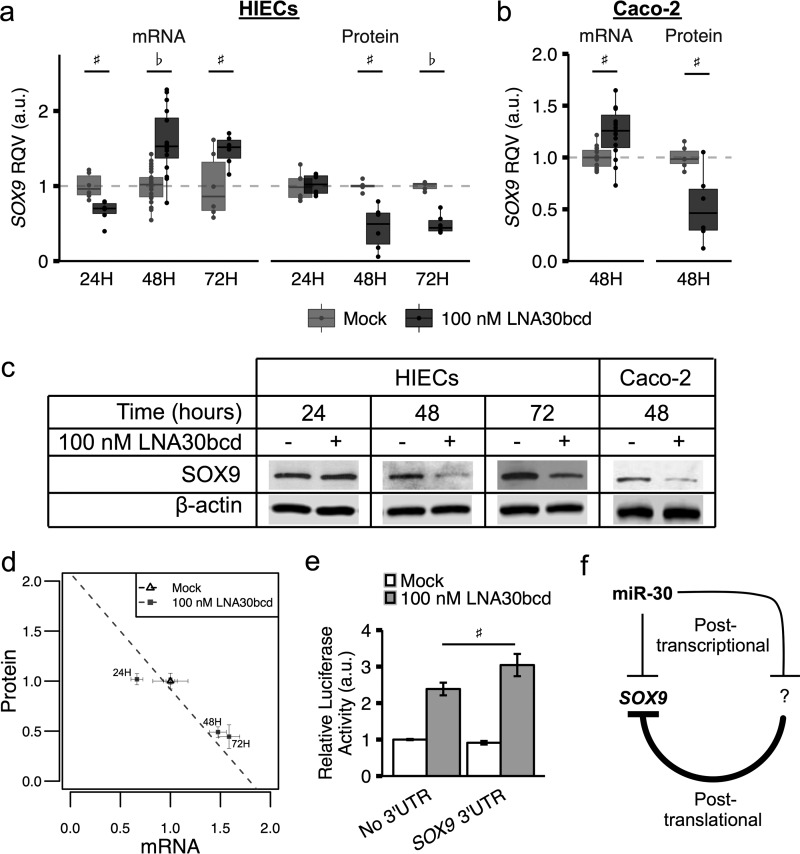
**Knockdown of miR-30 increases *SOX9* mRNA and decreases SOX9 protein expression.** *a*, relative quantitative value (RQV), across three time points, of *SOX9* mRNA by RT-PCR (*left panel*) and protein by Western blot densitometry (*right panel*) in HIECs upon either mock transfection or 100 nm LNA30bcd transfection. *b*, RQV of *SOX9* mRNA by RT-PCR (*left panel*) and protein by Western blot densitometry (*right panel*) in Caco-2 cells upon mock transfection or 100 nm LNA30bcd transfection after 48 h (mock and 100 nm LNA30bcd mRNA *n* = 15 each, protein *n* = 6 each). *c*, images of representative Western blots are shown for the protein expression data shown in *a* and *b. d*, correlation of mean RQVs of SOX9 mRNA and protein across time points and transfection conditions. *e*, relative firefly luciferase activity in Caco-2 cells at 48 h after transfection with plasmids encoding both firefly (with and without the *SOX9* 3′-UTR) and *Renilla* luciferase genes. Caco-2 cells were subjected to either mock co-transfection or 100 nm LNA30bcd co-transfection (*n* = 10–11 each). *f*, model of miR-30 regulation of SOX9 in the intestinal epithelium. For *a* and *b*, standard box and whisker plots are shown, with the *shaded boxes* indicating inner quartile ranges (IQR), the *thick horizontal line* showing median, and extending *whiskers* showing maximum and minimum points within 1.5*IQR. Actual data points are plotted as *filled circles* superimposed on their respective box and whisker plots. ♭, *p* < 0.001; #, *p* < 0.05.

To evaluate miR-30 regulation of *SOX9* in IECs, we knocked down miR-30 expression using locked nucleic acids complementary to miR-30b, miR-30c, and miR-30d (LNA30bcd), in human intestinal epithelial cells (HIECs). Upon knockdown of these miR-30 family members, we observed a significant increase in *SOX9* mRNA at 48 and 72 h post-transfection (Fig. 2*a*), which is consistent with alleviation of negative post-transcriptional regulation of *SOX9* by miR-30. However, we unexpectedly found that SOX9 protein was significantly down-regulated (Fig. 2, *a* and *b*). In fact, *SOX9* mRNA and protein expression were strongly inversely correlated (Pearson's *r* = −0.93, *p* = 0.006; Fig. 2*a*) across three time points post-transfection with LNA30bcd. We confirmed that this inverse relationship between *SOX9* mRNA and protein exists in a second intestinal cell culture model, Caco-2 (Fig. 2*b*), indicating that the finding is not unique to HIECs. To test for a direct relationship between miR-30 and the *SOX9* 3′-UTR, we performed a luciferase reporter assay in Caco-2 cells. We observed increased relative luciferase activity in cells transfected with 100 nm LNA30bcd (Fig. 2*d*), consistent with direct targeting of *SOX9* by miR-30 that has been previously shown in cartilage (35). We hypothesized that the opposite effect of miR-30 inhibition on SOX9 mRNA and protein levels could be due to miR-30-mediated regulation of factors that modify SOX9 protein stability without affecting *SOX9* RNA levels, such as post-translational modifiers (Fig. 2*e*).

##### Next Generation High Throughput RNA Sequencing Reveals That miR-30 Regulates Genes Enriched in the Ubiquitin Ligase Pathway

**FIGURE 3.**
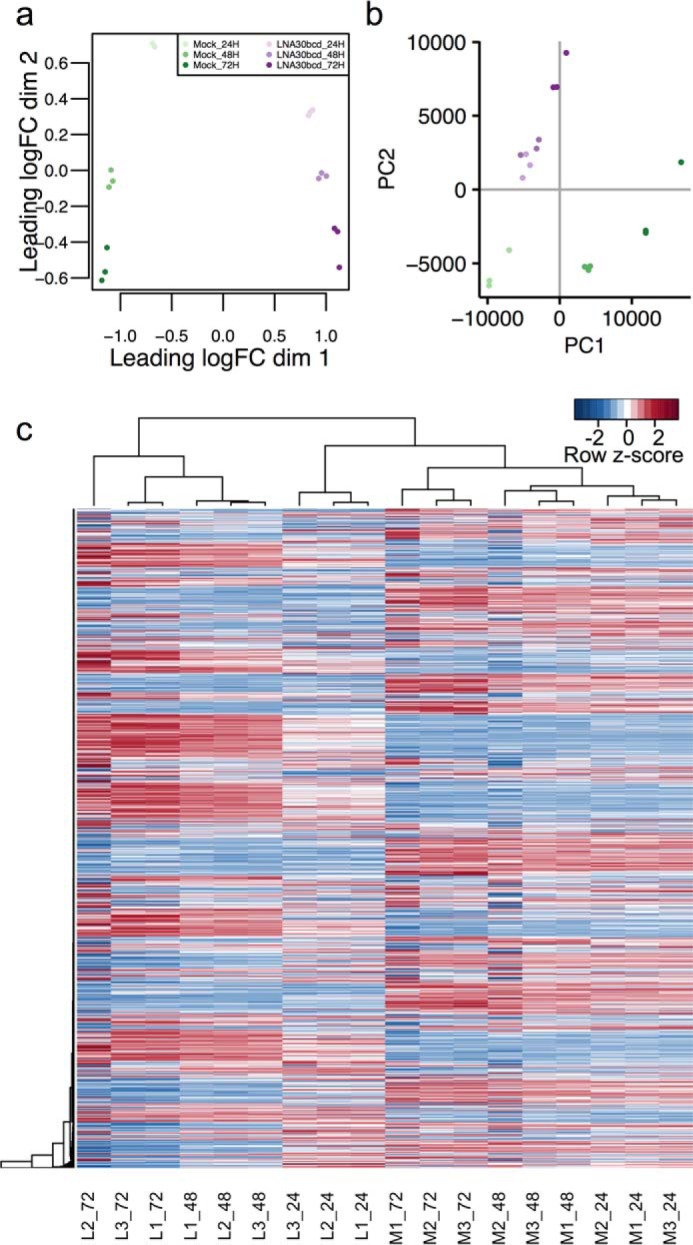
**Next generation high throughput RNA sequencing of LNA30bcd-treated HIECs.** *a*, multidimensional scaling plot of normalized reads (counts per million or CPM > 10 in ≥3 samples) in HIECs subjected to either mock transfection or 100 nm LNA30bcd transfection at 24 h (*24H*), 48 h (*48H*), and 72 h (*72H*) post-transfection (*n* = 3 each). *b*, principle components analysis of normalized reads (CPM > 10 in 3+ samples) across all time points and transfection conditions. *c*, heat map showing all genes with CPM > 10 in ≥3 samples (*n* = 10,096). Samples are hierarchically clustered by Euclidean distance. For each column, samples are listed along the bottom, with the first letter indicating mock treated (*M*) or 100 nm LNA30bcd-treated (*L*) HIECs, followed by the replicate number (replicate 1, 2, or 3), and the time point post-transfection (24, 48, or 72 h).

**FIGURE 4.**
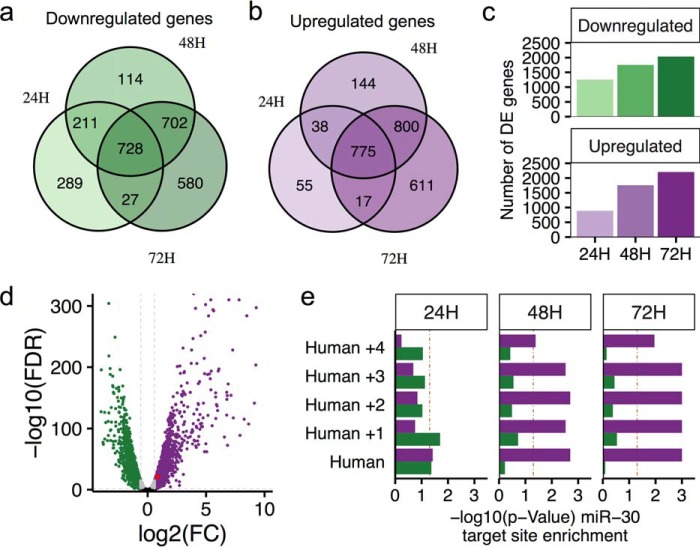
**LNA30bcd-treated HIECs undergo robust changes in gene expression over a 3-day time course.** *a*, Venn diagram showing significantly down-regulated genes (fold change or FC < −1.5, FDR < 0.05) across time points in HIECs after 100 nm LNA30bcd transfection compared with mock transfection. *b*, Venn diagram showing significantly up-regulated genes (FC > 1.5, FDR < 0.05) across time points in HIECs after 100 nm LNA30bcd transfection compared with mock transfection. *c*, bar graph showing the number of significantly down-regulated and up-regulated genes across time points. *d*, volcano plot showing differentially expressed genes at 72 h. The *red dot* shows *SOX9*. The *horizontal dashed line* shows *p* = 0.05, and *vertical dashed lines* indicate FC = −1.5 and 1.5. *e*, results of miRhub analysis to test for enrichment of predicted miR-30 target sites in significantly up-regulated (*purple*) and down-regulated (*green*) genes at each time point. Our analysis was human-centric. Each row indicates the conservation of the miRNA target site on the gene list, with *Human* indicating a site found in human genes and *Human* +*1* indicating a site found in human genes and conserved in one additional species, and so on. The *vertical dashed line* (*red*) indicates empirical *p* = 0.05.

To evaluate this hypothesis, we next sought to define the regulatory program that miR-30 directs in HIECs and to identify potential miR-30 targets that may be regulating SOX9 protein levels. Specifically, we performed next generation high throughput RNA sequencing on total RNA isolated from mock and LNA30bcd transfected HIECs at three time points (see “Experimental Procedures”). Following read alignment and transcript quantification, we identified differentially expressed genes using edgeR (36). To avoid bias from lowly expressed genes, we filtered out genes that did not reach an expression threshold of 10 counts per million (CPM) in at least three of the samples. A total of 10,096 genes were included in our analysis. We first normalized gene counts using the generalized linear model in edgeR to account for both the treatment and time variables in our experimental design. Samples were tightly clustered by treatment and time point according to multidimensional scaling, principal components, and hierarchical clustering analyses (Fig. 3, *a–c*). Notably, cells treated with 100 nm LNA30bcd at 24 h post-transfection clustered with mock transfected samples (Fig. 3*c*). However, cells treated with 100 nm LNA30bcd at 48 and 72 h post-transfection clustered into a distinct subclade, indicating that the regulatory effect of LNA30bcd was greatest at the later time points. Next, we performed differential expression analysis and found that half of all genes (50.1% or 5055) included in the analysis are significantly differentially expressed (FC ± 1.5 and FDR < 0.05) between mock and LNA30bcd transfected cells in at least one time point post-transfection (Fig. 4, *a–c*, and supplemental Table S1). Notably, although *SOX9* was found to be up-regulated as expected by LNA30bcd treatment at 72 h post-transfection, it was certainly not the most robustly or significantly altered gene (Fig. 4*d*).

To evaluate the efficacy of our knockdown, we performed analysis with miRhub (37–39), which tests for miRNA target site enrichment among specific genes of interest. We found that both highly conserved and species-specific predicted miR-30 targets sites were significantly enriched (*p* < 0.05) in genes up-regulated at both 48 and 72 h post-transfection, but as expected not in down-regulated genes (Fig. 4*e*). At 24 h post-transfection, predicted miR-30 target sites were not enriched. Together, these data suggest that our knockdown of miR-30 using LNA30bcd was specific and highly effective in HIECs, particularly in the later time points of our study.

**FIGURE 5.**
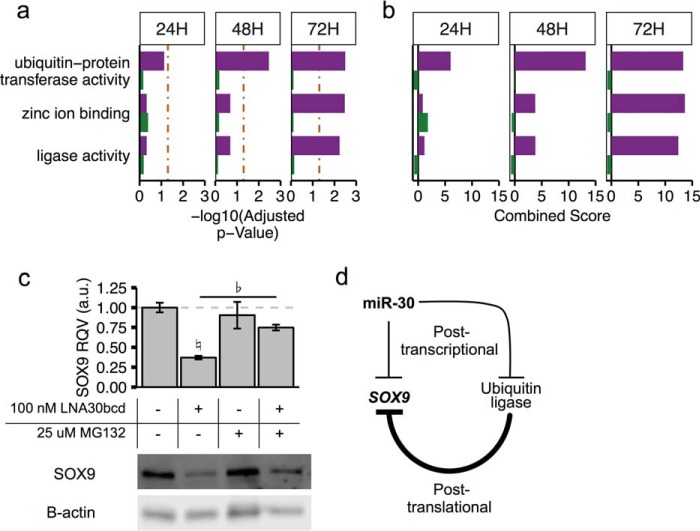
**miR-30 target genes in intestinal epithelial cells are over-represented in the ubiquitin ligase pathway.** *a* and *b*, Gene Ontology Molecular Function enrichment analysis using Enrichr identifies three terms as enriched in the up-regulated gene lists across time points. *p* value (*a*) and combined scores (*b*) shown. *c*, relative SOX9 protein expression in Caco-2 cells subject to mock transfection (*n* = 2 each) or 100 nm LNA30bcd transfection (*n* = 3 each) at 72 h post-transfection following 4 h of treatment with either 25 μm MG132 or vehicle (DMSO). The *bottom panel* shows a representative Western blot. *d*, cartoon showing model of miR-30 regulation of SOX9 mRNA and protein expression levels.

To identify genes that might act as post-translational regulators of SOX9 protein in response to LNA30bcd treatment, we performed Gene Ontology Molecular Function enrichment analysis (40, 41) using Enrichr (42) on genes with predicted miR-30 target sites that were significantly up-regulated (FC > 1.5 and FDR < 0.05) relative to mock treated cells at each time point (see supplemental Table S2 for gene lists). Only three terms were identified as being significantly enriched (adjusted *p* value < 0.05; Fig. 5, *a* and *b*) at any time point in the up-regulated gene sets. Interestingly, these included “ubiquitin-protein transferase activity” and “ligase activity.” Ubiquitin ligase-mediated regulation of SOX9 has been shown previously in chondrocytes (43) and therefore is consistent with our hypothesis that miR-30 may regulate SOX9 protein levels indirectly through control of post-translational modifiers of SOX9.

To evaluate whether miR-30 influences ubiquitin ligase-mediated degradation of SOX9 protein, we subjected Caco-2 cells to either mock or LNA30bcd transfection and then treated them with vehicle or MG132, a potent proteasome inhibitor. We found that MG132 treatment for 4 h was sufficient to rescue SOX9 protein expression following LNA30bcd treatment (Fig. 5*c*). This suggests that miR-30 is able to regulate SOX9 protein expression through post-transcriptional regulation of ubiquitin ligases (Fig. 5*d*).

##### miR-30 Promotes IEC Proliferation and Inhibits IEC Differentiation

**FIGURE 6.**
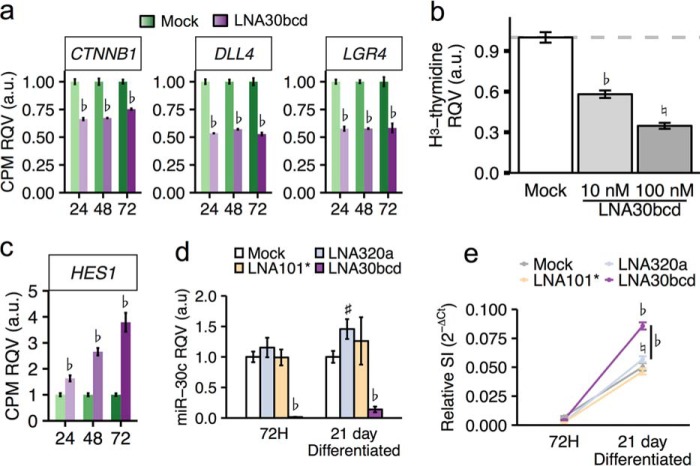
**miR-30 promotes proliferation and inhibits enterocyte differentiation.** *a*, RQV of mean CPM of markers of proliferation in 100 nm LNA30bcd-treated HIECs across time points. Significance was determined by using edgeR generalized linear model accounting for treatment and time. ♭, FDR < 0.001; ♮, FDR < 0.01; #, FDR < 0.05. *b*, RQV of [^3^H]thymidine incorporation in HIECs subjected to mock transfection or LNA30bcd transfection (10 or 100 nm) at 48 h post-transfection (*n* = 12 each). Significance was determined by two-tailed unpaired Student's *t* test. ♭, *p* < 0.001; ♮, *p* < 0.01; #, *p* < 0.05. *c*, RQV of mean CPM of *HES1* (Hairy and Enhancer of Split 1) in 100 nm LNA30bcd-treated HIECs across time points (*n* = 3 each). Significance was determined by generalized linear model accounting for treatment and time. ♭, FDR < 0.001; ♮, FDR < 0.01; #, FDR < 0.05. *d*, RQV of miR-30c by RT-PCR in Caco-2 cells at 72 h and 21 days after either mock transfection (72 h, *n* = 9; 21 days, *n* = 6), 100 nm LNA30bcd transfection (72 h, *n* = 9; 21 days *n* = 6), 100 nm LNA101* (72 h, *n* = 6; 21 days, *n* = 3), or 100 nm LNA320a (72 h, *n* = 9; 21 days, *n* = 6). Significance was determined using two-tailed unpaired Student's *t* test. ♭, *p* < 0.001; ♮, *p* < 0.01; #, *p* < 0.05. *e*, RQV of sucrase isomaltase (*SI*) by RT-PCR in in Caco-2 cells at 72 h and 21 days after either mock transfection (72 h, *n* = 9; 21 days *n* = 8), 100 nm LNA30bcd (72 h, *n* = 9; 21 days, *n* = 9), 100 nm LNA101* (72 h, *n* = 6; 21 days, *n* = 6), or 100 nm LNA320a (72 h, *n* = 9; 21 days, *n* = 9). Significance was determined using two-tailed unpaired Student's *t* test. ♭, *p* < 0.001; ♮, *p* < 0.01; #, *p* < 0.05.

Based on previous work, altered levels of SOX9 are expected to lead to changes in the balance between proliferation and differentiation (15, 16, 44). Therefore, given the strong regulatory effect of miR-30 on SOX9 protein, we hypothesized that treatment of HIECs with LNA30bcd would affect this balance as well. Notably, we found by analysis of the RNA sequencing data that the expression of genes previously associated with proliferation in the intestinal epithelium (Fig. 6*a*), including *CTNNB1* (45), *DLL4* (46), and *LGR4* (47), were significantly reduced. Consistent with this observation, we found that knockdown of miR-30 significantly reduces HIEC proliferation, as measured by [^3^H]thymidine uptake (Fig. 6*b*). At 48 h post-transfection, HIECs showed a 65% reduction in [^3^H]thymidine uptake after treatment with 100 nm of LNA30bcd (*p* < 0.001; Fig. 6*b*).

Given the reduced proliferation, we hypothesized that treatment with LNA30bcd may promote differentiation of IECs. We evaluated differential expression of genes known for their role in regulating differentiation in the intestinal epithelium using the RNA sequencing data. Interestingly, we observed a 5-fold increase in *HES1* expression in HIECs transfected with 100 nm LNA30bcd (Fig. 6*b*). *HES1* is an early marker of enterocyte differentiation in the intestinal epithelium (48). The Caco-2 cell line is one of very few cell models that will spontaneously differentiate into small intestinal enterocyte-like cells and express key markers of mature enterocytes upon reaching confluency (49, 50). To test whether miR-30 regulates enterocyte differentiation of IECs, we transfected Caco-2 cells with 100 nm LNA30bcd and allowed the cells to differentiate on Transwell membranes (see “Experimental Procedures”). With a single transfection of LNA30bcd, we observed significant and sustained knockdown of miR-30 levels for 21 days, the latest time point measured (Fig. 6*d*). At 21 days post-transfection, we also observed that Caco-2 cells transfected with LNA30bcd expressed significantly higher levels of sucrose isomaltase, a classic marker of differentiated enterocytes (51), compared with mock transfected cells or those transfected with LNAs against other miRNAs (Fig. 6*e*). Taken together, our data suggest that miR-30 normally acts to promote proliferation and inhibit enterocyte differentiation in the intestinal epithelium through a broad regulatory program that includes the proteasome pathway.

## Discussion

In this study, we sought to investigate miRNA control of intestinal epithelial proliferation and differentiation. Our starting point was to identify miRNAs that might regulate SOX9, a key transcription factor in intestinal epithelial homeostasis. We focused on miR-30 because it has a *SOX9* target site that is broadly conserved across vertebrates, including human and rodent, and it is robustly and variably expressed among stem, progenitor, and differentiated cell types of the intestinal epithelium. Upon knockdown of miR-30 in two intestinal-relevant cell lines, we unexpectedly found inverse effects on SOX9 mRNA and protein expression. We performed next generation high throughput RNA sequencing and found that up-regulated genes with predicted miR-30 target sites were most significantly enriched for ubiquitin ligases. Post-translation regulation of SOX9 by UBE3A has been described previously (43). Moreover, UBE3A does have a predicted miR-30 target site and is up-regulated in LNA30bcd-treated HIECS. However, the predicted miR-30 target site in *UBE3A* is human-specific. It is therefore possible that the inverse relationship between *SOX9* mRNA and protein in response to LNA30bcd treatment is human-specific. More research will be needed to identify the specific miR-30-directed ubiquitin ligase protein that acts on SOX9 protein in intestinal epithelial cells.

Knockdown of the miR-30 family in HIECs and Caco-2 cells resulted in reduced proliferation and enhanced enterocyte differentiation. This finding is consistent with the relatively higher expression levels of miR-30 in proliferating subpopulations, such as the progenitors, compared with non-proliferating enterocytes (Fig. 1*b*). Previous literature investigating the role of miR-30 suggests a dosage- and cell type-specific response on proliferation. Although increased proliferation has been seen in many cancer cells in response to reduced miR-30 levels, a number of studies have found knockdown of miR-30 to result in decreased proliferation (52). In terms of differentiation, the miR-30 family has been shown to regulate myogenic and osteoblastic differentiation. Up-regulation of miR-30 family members in myoblasts promotes differentiation (53). Alternatively, knockdown of miR-30 in an osteoblast precursor cell line promotes differentiation (54). Our results, and those of previous studies, emphasize the importance of conducting cell type-specific analyses on miRNA regulatory networks. Moreover, further research is warranted to evaluate miR-30 regulatory networks in the intestinal epithelium *in vivo*.

More broadly, our RNA sequencing revealed a complex and widespread network of genes influenced by knockdown of a single miRNA family. Through time course mRNA profiling following knockdown of a single miRNA family, we found that the effect of treatment with LNA30bcd on miR-30 target genes was only beginning to emerge at 24 h, evident at 48 h, and very robust at 72 h post-transfection. Most studies using LNAs against target miRNAs evaluate knockdown and gene expression changes at a single time point post-transfection. It is clear from our data that there are highly variable effects of miRNA knockdown across a span of only 2 days, emphasizing the importance of evaluating multiple time points following treatment with LNAs. In Caco-2 cells we observed significant knockdown of miR-30 even 21 days following a single transfection with LNA30bcd; therefore, it would of interest to evaluate gene expression at this time point to determine whether the effects on miR-30 target genes are still robust. Detailed time course studies may help elucidate the short term and long term effects of LNA treatment, which has relevance both for experimental design and for therapeutic development.

Our analyses provide new evidence that miR-30 plays a significant role in regulating proliferation and differentiation in the intestinal epithelium. Further analyses *in vivo* (mouse) or through *ex vivo* culture systems (mouse or human) are warranted to extend the definition of the function of miR-30 across distinct cell types of the intestinal epithelium in health and disease. This study represents one of the very first to investigate the regulatory activity of a specific miRNA in intestinal epithelial cells using a highly interdisciplinary strategy and therefore provides a blueprint for similar studies of other miRNAs.

## Experimental Procedures

### 

#### 

##### Animals

All animal studies were approved by the University of North Carolina at Chapel Hill Institutional Animal Care and Use Committee (protocol 13-162). *Sox9-EGFP* female mice (20–22) on a CD1 background were fed a standard chow diet (Prolab RMH3000) *ad libitum*. Eleven-week-old mice were euthanized with a lethal dose of Nembutal (150 μg/gram of body weight) and were processed for jejunal IEC dissociation and fluorescence-activated cell sorting.

##### IEC Dissociation for Flow Cytometry and FACS

The small intestine was dissected and flushed with ice-cold PBS to remove contents and then divided into three equal sections. The middle section was considered jejunum and was prepared for FACS as previously described (21, 23). IECs were sorted using a Mo-Flo XDP cell sorter (Beckman-Coulter, Fullerton, CA) at the University of North Carolina Flow Cytometry Core Facility using previously described gating parameters (20, 21, 23). Cells that stained for CD31 (BioLegend, San Diego, CA), CD45 (BioLegend, San Diego, CA), and or annexin V (Life Technologies), were excluded prior to sorting. Following sorting, cells were pelleted by centrifugation, and total RNA was isolated using the Norgen Total RNA kit (Norgen Biotek, Thorold, Canada) as per the manufacturer's instructions. Nanodrop 2000 was used to quantify RNA.

##### Quantitative RT-PCR

TaqMan microRNA reverse transcription kit (Applied Biosystems) and high capacity RNA to cDNA kit were used as per the manufacturers' instructions to generate complementary DNA for miRNA and gene expression assays, respectively. RT-PCR was performed using TaqMan Universal PCR Master Mix (Applied Biosystems) for miRNA RT-PCR and Gene Expression Master Mix (Applied Biosystems) for mRNA quantification. RT-PCR assays were run on a Bio-Rad CFX96 Touch real time PCR detection system (Bio-Rad). The assays were performed in triplicate using either *U6* (miRNA expression) or *RPS9* (mRNA expression) as an internal control. All TaqMan assays were purchased from Applied Biosystems and include: miR-30a (assay 000417), miR-30b (assay 000602), miR-30c (assay 000419), miR-30d (assay 000420), miR-30e (assay 002223), miR-101a (assay 002253), miR-101b (assay 002531), miR-320a (assay 002277), miR-145 (assay 000467), *U6* (assay 001973), *Sox9* (assay Mm00448840_m1), *Rps9* (assay Mm00850060_s1), *SOX9* (assay Hs01001343_g1), *HES1* (assay Hs00172878_m1), sucrose isomaltase (assay Hs00356112_m1), and *RPS9* (assay Hs02339424_g1).

##### Cell Culture and Transfections

HIECs were acquired from the Beaulieu laboratory (55) and were cultured in OptiMEM 1 (Life Technologies) supplemented with 10% FBS (Life Technologies), 0.01 m HEPES (Life Technologies), and 5 ng/ml hEGF (Invitrogen). The cells were used between passages 20 and 30 and were maintained at 70% confluency. HIECs were seeded onto tissue-culture treated plates and transfected at 70% confluency with 3.25 μl/ml Lipofectamine 2000 (Life Technologies).

*Caco-2* cells were cultured in high glucose DMEM (Sigma-Aldrich) supplemented with 10% FBS. The cells were used between passages 18 and 30 and were maintained at 70% confluency. Caco-2 cells were seeded onto tissue-culture treated plates and transfected at 70% confluency with 1.875 μl/ml Lipofectamine 3000 (Life Technologies).

Locked Nucleic Acids were purchased from Exiqon (Woburn, MA) including hsa-miR-101* (catalog no. 4101585–101), mmu-miR-30bcd (catalog no. 199900), and hsa-miR-320a (catalog no. 4101458–101). LNAs against mouse miR-30 family members are cross-reactive with the human miR-30 family.

For MG132 treatment studies, 6 μl of 10 mm MG132 (Z-Leu-Leu-Leu-al; Sigma-Aldrich, catalog no. C2211) or DMSO vehicle was added to each well of a 6-well plate for a final concentration of 25 μm MG132 at 68 h post-transfection. Following a 4-h treatment, the cells were isolated for RNA and protein as described below.

##### Caco-2 Differentiation

Similar to methods previously described (24, 56, 57), Caco-2 cells between passages 23 and 27 were grown on 100-mm tissue culture-treated plates (Corning catalog no. 430167). At 70% confluency, cells were transfected with 100 nm LNA against miR-30bcd, miR-320a, or miR-101*. At 24 h post-transfection, the cells were trypsinized, and 2 × 10^5^ cells were seeded onto Transwell inserts (Costar catalog no. 3460; Fisher Scientific). Reseeding onto the Transwells following transfection was done to avoid differences in cell density caused by cell death or changes in cell proliferation following transfection with each LNA. Differentiation was monitored every other day using transepithelial electric resistance beginning at 72 h post-transfection. The cells were considered fully differentiated after 1 week following the beginning of the transepithelial electric resistance plateau (58). Throughout differentiation, the medium was changed from both the top and bottom wells every other day following transepithelial electric resistance measurement. At 72 h post-transfection, undifferentiated cells were harvested for RNA. At 21 days post-transfection, differentiated cells were harvested for RNA.

##### Western Blot

Protein was isolated from cells as previously described (38) and was quantified using the Pierce Microplate BCA protein assay kit - reducing agent compatible (Thermo-Scientific), run on Bio-Rad Any-kDa Mini-Protean TGX precast gels, and transferred to nitrocellulose membranes in the Bio-Rad Midi Transfer Packs using the Bio-Rad Trans-Blot turbo blotting system. The membranes were blocked 1 h in 5% milk, before being probed overnight at 4 °C with SOX9 antibody (1:800, Abcam catalog no. ab26414). Secondary antibody was applied for 2 h following wash steps at the following dilutions: goat α-rabbit (1:4000, Abcam ab97069). Preconjugated β-actin-HRP (1:40,000, Sigma-Aldrich catalog no. A3854) was applied for 20 min and used as loading control. Western blot densitometry analysis was done using ImageJ.

##### RNA Sequencing

Total RNA from mock and miR-30bcd LNA-treated HIECs were isolated at 24, 48, and 72 h post-transfection. RNA quality was assessed using Agilent RNA Nano 6000 kit (Agilent Technologies, Inc, Santa Clara, CA) and then run on a Bioanalyzer 2100 (Agilent). All samples had high RNA integrity numbers, with RNA integrity numbers above 9.2 (with an average of 9.7). Samples were submitted to the University of North Carolina High Throughput Sequencing Facility for TruSeq Stranded Total RNA library preparation (Illumina, San Diego, CA) and paired end 50-bp sequencing on a HiSeq 2000 (Illumina) multiplexing 6 samples/lane.

##### Bioinformatics

Following sequencing and demultiplexing by the University of North Carolina High Throughput Sequencing Facility, reads were aligned to the hg19 genome using MapSplice (59), and transcripts were quantified using RSEM (60) by the University of North Carolina Bioinformatics Core Facility. Samples had an average of 119 million reads, with 94.6% of reads uniquely mapping. Differential gene expression analysis was conducted using edgeR (36). Genes with low expression (CPM < 10 in more than half the samples) were filtered out of our analysis. Gene counts were then normalized using trimmed mean of the M-values (TMM) method and evaluated for differential gene expression. Raw sequencing data, as well as the raw and normalized count tables, are available through GEO (accession no. GSE79923).

## Author Contributions

B. C. E. P. conceptualized experiments, conducted or supervised all experiments, analyzed and interpreted data, prepared figures, and drafted and revised the manuscript. A. T. M. provided assistance with animal experiments including colony maintenance and the preparation and sorting of cells from the Sox9-EGFP animals. J. S. and S. F. conducted experiments under the supervision and guidance of B. C. E. P. J. G. S. assisted with experiments using radiolabeled [^3^H]thymidine. P. K. L. provided assistance with conceptualization, experimental design, and interpretation. P. S. obtained funding, conceptualized experiments and experimental design, interpreted data, supervised the overall study, and revised the manuscript.

## Supplementary Material

Supplemental Data

## Footnotes

2 The abbreviations used are:

miRNA: microRNA
IEC: intestinal epithelial cell
LNA: locked nucleic acid
HIEC: human intestinal epithelial cell
CPM: counts per million
FC: -fold change
FDR: false discovery rate
RQV: relative quantitative value.
