# Supplementary material for: miR-30 Family Controls Proliferation and Differentiation of Intestinal Epithelial Cell Models by Directing a Broad Gene Expression Program That Includes SOX9 and the Ubiquitin Ligase Pathway
Source: J Biol Chem. 2016 Jun 3;291(31):15975–84. doi: 10.1074/jbc.M116.733733 (PMC4965549; doi:10.1074/jbc.M116.733733)
Supplement: Supplemental Data [file supp_291_31_15975__index.html]

miR-30 family controls proliferation and differentiation of intestinal epithelial cell models by directing a broad gene expression program that includes SOX9 and the ubiquitin ligase pathway — miR-30 Family Controls Proliferation and Differentiation of Intestinal Epithelial Cell Models by Directing a Broad Gene Expression Program That Includes SOX9 and the Ubiquitin Ligase Pathway — miR-30 in Intestinal Epithelial Homeostasis — Supplemental Data 

# miR-30 Family Controls Proliferation and Differentiation of Intestinal Epithelial Cell Models by Directing a Broad Gene Expression Program That Includes SOX9 and the Ubiquitin Ligase Pathway

## Supplemental Data

- Supplemental Table 1: Differential gene expression data (.xlsx, 957 KB) - Differential gene expression analysis using edgeR of RNA-sequencing data in LNA30bcd treated human intestinal epithelial cells (HIECs), across three time points. Data for each time point is provided as an individual TAB in the XLSX file.
- Supplemental Table 2 (.xlsx, 283 KB) - Differentially expressed predicted miR-30 target genes for each time point are provided in TAB 1. Subsequent TABs contain Gene Ontology Molecular Function Enrichment analysis data generated using Enrichr.
